#  Trust in Physicians and Hospitals During the COVID-19 Pandemic in a 50-State Survey of US Adults

**DOI:** 10.1001/jamanetworkopen.2024.24984

**Published:** 2024-07-31

**Authors:** Roy H. Perlis, Katherine Ognyanova, Ata Uslu, Kristin Lunz Trujillo, Mauricio Santillana, James N. Druckman, Matthew A. Baum, David Lazer

**Affiliations:** 1 Center for Quantitative Health, Massachusetts General Hospital, Boston, Massachusetts; 2 Department of Psychiatry, Harvard Medical School, Boston, Massachusetts; 3 Associate Editor, *JAMA Network Open*; 4 Department of Communication, School of Communication and Information, Rutgers University, New Brunswick, New Jersey; 5 Department of Political Science, Northeastern University, Boston, Massachusetts; 6 Department of Political Science, University of South Carolina, Columbia; 7 Department of Political Science, University of Rochester, Rochester, New York; 8 John F. Kennedy School of Government and Department of Government, Harvard University, Cambridge, Massachusetts

## Abstract

**Importance:**

Trust in physicians and hospitals has been associated with achieving public health goals, but the increasing politicization of public health policies during the COVID-19 pandemic may have adversely affected such trust.

**Objective:**

To characterize changes in US adults’ trust in physicians and hospitals over the course of the COVID-19 pandemic and the association between this trust and health-related behaviors.

**Design, Setting, and Participants:**

This survey study uses data from 24 waves of a nonprobability internet survey conducted between April 1, 2020, and January 31, 2024, among 443 455 unique respondents aged 18 years or older residing in the US, with state-level representative quotas for race and ethnicity, age, and gender.

**Main Outcome and Measure:**

Self-report of trust in physicians and hospitals; self-report of SARS-CoV-2 and influenza vaccination and booster status. Survey-weighted regression models were applied to examine associations between sociodemographic features and trust and between trust and health behaviors.

**Results:**

The combined data included 582 634 responses across 24 survey waves, reflecting 443 455 unique respondents. The unweighted mean (SD) age was 43.3 (16.6) years; 288 186 respondents (65.0%) reported female gender; 21 957 (5.0%) identified as Asian American, 49 428 (11.1%) as Black, 38 423 (8.7%) as Hispanic, 3138 (0.7%) as Native American, 5598 (1.3%) as Pacific Islander, 315 278 (71.1%) as White, and 9633 (2.2%) as other race and ethnicity (those who selected “Other” from a checklist). Overall, the proportion of adults reporting a lot of trust for physicians and hospitals decreased from 71.5% (95% CI, 70.7%-72.2%) in April 2020 to 40.1% (95% CI, 39.4%-40.7%) in January 2024. In regression models, features associated with lower trust as of spring and summer 2023 included being 25 to 64 years of age, female gender, lower educational level, lower income, Black race, and living in a rural setting. These associations persisted even after controlling for partisanship. In turn, greater trust was associated with greater likelihood of vaccination for SARS-CoV-2 (adjusted odds ratio [OR], 4.94; 95 CI, 4.21-5.80) or influenza (adjusted OR, 5.09; 95 CI, 3.93-6.59) and receiving a SARS-CoV-2 booster (adjusted OR, 3.62; 95 CI, 2.99-4.38).

**Conclusions and Relevance:**

This survey study of US adults suggests that trust in physicians and hospitals decreased during the COVID-19 pandemic. As lower levels of trust were associated with lesser likelihood of pursuing vaccination, restoring trust may represent a public health imperative.

## Key Points

**Question:**

How did trust in physicians and hospitals change during the COVID-19 pandemic?

**Findings:**

In every sociodemographic group in this survey study among 443 455 unique respondents aged 18 years or older residing in the US, trust in physicians and hospitals decreased substantially over the course of the pandemic, from 71.5% in April 2020 to 40.1% in January 2024. Individuals with lower levels of trust were less likely to have been vaccinated or received boosters for COVID-19.

**Meaning:**

This study suggests that the COVID-19 pandemic has been associated with a continuing decrease in trust in physicians and hospitals, which may necessitate strategies to rebuild that trust to achieve public health priorities.

## Teaser

This survey study examines changes in US adults’ trust in physicians and hospitals over the course of the COVID-19 pandemic and the association between this trust and health-related behaviors.

## Introduction

Physicians have traditionally represented a key element in public health outreach efforts; most adults will see a physician on a regular basis, and these appointments represent an opportunity to encourage healthy behaviors ranging from diet and exercise^1^ to smoking cessation^2^ to seatbelt use^3^ and firearm safety.^4^ A 2022 survey reported that US adults had greater trust in physicians and nurses than in any other institution, including the Centers for Disease Control and Prevention,^5^ a result supported by a Kaiser Family Foundation tracking poll of 2007 adults conducted in 2023.^6^ Levels of trust increased early in the COVID-19 pandemic; in a Gallup poll, belief that physicians had high or very high ethical standards increased from 65% in 2019 to 77% in 2020.^7^

However, undercurrents of distrust in medicine are not new in US society—for example, concerns about the health effects of vaccines have persisted long after they were disproven.^8^ During the COVID-19 pandemic, medicine and public health more broadly became politicized, with the internet amplifying public figures^9^ and even physicians^10^ encouraging individuals not to trust the advice of public health experts and scientists.^11^ As such, the pandemic may have represented a turning point in trust, with a profession previously seen as trustworthy increasingly subject to doubt. By 2023, 1 poll showed perception of physician ethics to be substantially below the prepandemic baseline.^7^

In the present study, we drew on a 50-state US survey that began early in the COVID-19 pandemic to seek to characterize change in trust in physicians and hospitals over the course of the pandemic, aiming to confirm that trust had decreased. We further investigated the extent to which trust in physicians and hospitals is associated with specific health behaviors, including vaccination and vaccine boosters, to assess the relevance of this trust to public health. As the survey also captured aspects of political preference, it allowed us to distinguish and control for a key potential source of confounding.

## Methods

We used data from 24 waves of a nonprobability internet survey conducted using a commercial vendor, PureSpectrum, which aggregates and deduplicates participants in multiple national panels. PureSpectrum is an online marketplace for survey panel samples working with multiple recruitment vendors. Each of those vendors incentivizes respondents for participation in surveys based on the length of the survey, their specific panelist profile, and target acquisition difficulty. The specific type of incentives for participation varies and may include cash, airline miles, gift cards, redeemable points, sweepstakes entrance, and vouchers. The survey was developed and overseen by a consortium of academic sites, the COVID States Project,^12^ formed early in the pandemic to understand COVID-19–related attitudes and behaviors. The survey was conducted approximately every 1 to 2 months beginning April 1, 2020, through January 31, 2024, among individuals aged 18 years or older residing in all 50 states and the District of Columbia. A full description of the survey waves and date ranges can be found in eTable 1 in Supplement 1. Participants provided informed consent online. The study protocol was reviewed and approved by the institutional review board of Harvard University as exempt as only deidentified data were used and no participant contact was required. This study followed the American Association for Public Opinion Research (AAPOR) reporting guideline.

To ensure representativeness of US adults, the survey used quotas for gender, age at first survey completion, and race and ethnicity within each state. We included attention checks and open-ended answers that were used to filter out unreliable or automated respondents (eTable 2 in Supplement 1). This nonprobability sampling approach has previously been shown to yield results that approximate those of probability-sampled designs and administrative data collection.^13,14^

Individual sociodemographic characteristics were self-reported. Race and ethnicity, as with gender and age, were collected to allow confirmation of the representativeness of the US population and reweighting of the sample. They were identified using a survey instrument that included categories for race and ethnicity. Information on trust in physicians and hospitals and trust in scientists was collected by asking, “How much do you trust the following people and organizations to do what is right?” followed by a list of entities with 4 choices (a lot, some, not too much, or not at all; eAppendix 2 in Supplement 1). In waves prior to August 2022, we asked a variant of this question, “How much do you trust the following people and organizations to do the right thing to handle the current coronavirus (COVID-19) outbreak?” We also asked about propensity to trust more generally, by asking “Generally speaking, would you say that most people can be trusted, or that you cannot be too careful in dealing with people? Please give your answer on a scale from 1 to 10, where 1 is you cannot be too careful and 10 is most people can be trusted.”

SARS-CoV-2 vaccination status in surveys after the availability of the vaccine was collected by asking individuals whether they had been vaccinated and later by number of prior vaccinations as a means of incorporating information about vaccine boosters. Influenza vaccination status was collected (in survey wave 27, from spring 2023) by asking about prior vaccination or intention to be vaccinated.

### Statistical Analysis

Survey results were reweighted with interlocking national weights for age at survey completion, gender, and race and ethnicity, as well as educational level and region, using 2019 US Census American Community Survey data,^15^ via the survey package in R, version 4.0 (R Project for Statistical Computing),^16^ a standard approach for nonprobability samples.^17^

We first used survey-weighted ordinal logistic regression to examine the association between physician trust score and a range of sociodemographic features, drawing on 2 of the survey waves; for these analyses, if a respondent completed both waves, we selected only the index response. We then applied survey-weighted logistic regression models with vaccination against SARS-CoV-2 or influenza as the outcome and incorporated trust in physicians and hospitals as well as sociodemographic features as independent variables. To examine the association between trust in physicians and hospitals reported on the prior survey wave and likelihood of vaccination against SARS-CoV-2 among individuals not previously vaccinated, we also used logistic regression, adjusting for the same covariates as in prior models. This analysis began with wave 18 (early summer 2021) as the first wave that occurred after vaccination was widely available in all US states. All *P* values were from 2-sided tests, and results were deemed statistically significant at *P* < .05.

On 1 survey wave (the first of the 2 examined cross-sectionally), we also asked the respondents an open-ended question to identify factors associated with different trust levels: “You said you trust doctors and hospitals [amount of trust]. Can you tell us why that is?” As standard topic modeling approaches do not perform well in brief text,^16^ we instead used a large language model to summarize themes and example text, to identify factors associated with poor trust (ie, the lowest level of trust). Specifically, we applied a large language model (Generative Pre-trained Transformer 4 turbo, gpt-4-1106-preview; OpenAI) with the temperature hyperparameter that controls the model’s randomness set at 0.7 to identify the 4 major themes reflected in the uncoded data from the open-ended responses. We used the following prompt: “Each of the following survey responses explains why someone does not trust doctors or hospitals. From them, without using any other knowledge, identify 4 main themes that can be explained in a brief phrase, plus an ‘other’ category for comments that don’t fit in any theme. Then provide 5 examples of responses in each category.” The model was presented with the full list of responses (via OpenAI’s Python API), then asked to characterize each response into the best-fitting category with a second prompt: “Please place each response in one of these categories that best fits,” to allow an estimate of the proportion reflected in each theme.

## Results

The combined data had 582 634 responses across 24 survey waves, including 443 455 unique respondents. The unweighted mean (SD) age was 43.3 (16.6) year; 288 186 (65.0%) reported female gender, and 155 269 (35.0%) reported male gender; 21 957 (5.0%) identified as Asian American, 49 428 (11.1%) as Black, 38 423 (8.7%) as Hispanic, 3138 (0.7%) as Native American, 5598 (1.3%) as Pacific Islander, 315 278 (71.1%) as White, and 9633 (2.2%) as other race and ethnicity (those who selected “Other” from a checklist).

**Figure 1.**
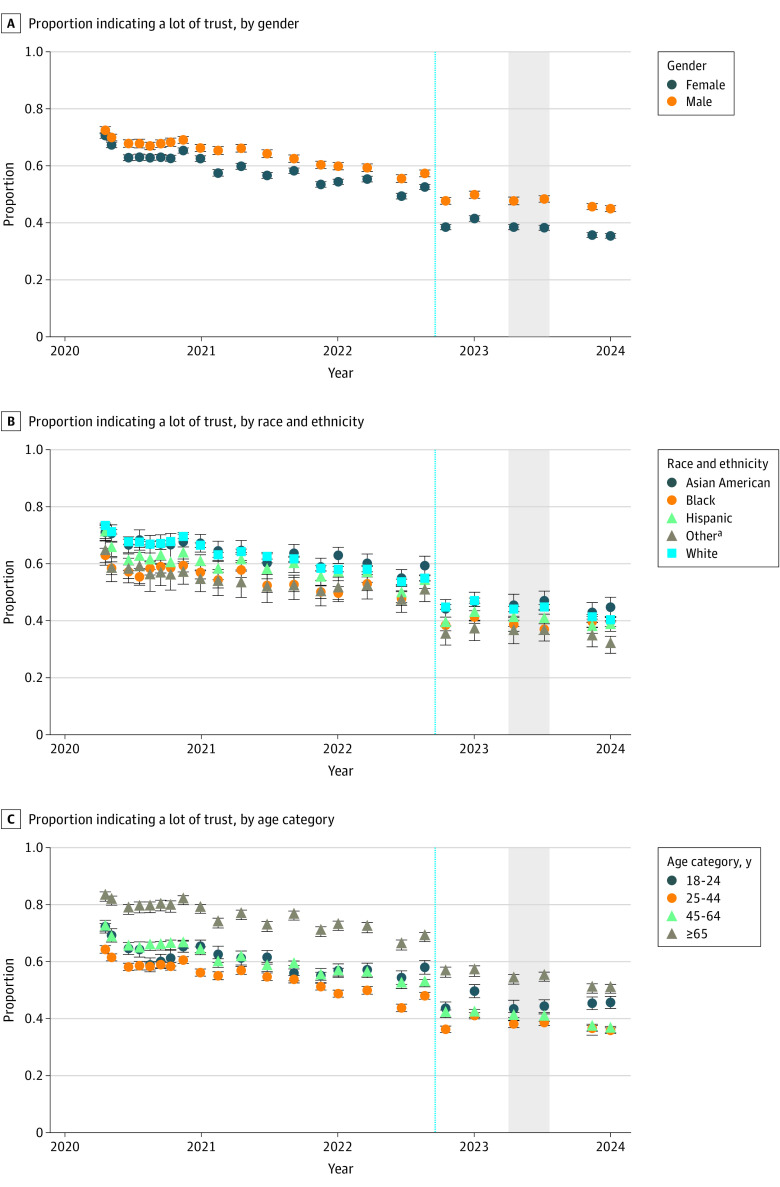
Trust in Physicians and Hospitals Over Time, Stratified by Gender, Race and Ethnicity, and Age Gray-shaded area spans surveys included in cross-sectional regression models. ^a^Other race and ethnicity refers to individuals who indicated Native American, Pacific Islander, or “Other” from a survey checklist.

Figure 1 illustrates the proportion of individuals who reported a lot of trust in physicians and hospitals in each survey wave over time, subdivided by gender, race and ethnicity, and age. Overall, the proportion of adults reporting a lot of trust for physicians and hospitals decreased from 71.5% (95% CI, 70.7%-72.2%) in April 2020 to 40.1% (95% CI, 39.4%-40.7%) in January 2024. (In light of the shift in wording for this question, we examined 4001 respondents from June and July 2023 randomly selected to answer both forms of the question; responses to the 2 versions were strongly correlated (Spearman ρ = 0.76; 95% CI, 0.74-0.78). eFigure 9 in Supplement 1 illustrates the change in proportion of responses at each level of trust at each survey wave.

We then focused on 2 waves in spring and summer 2023 (from April 5 to May 5, 2023, and from June 29 to August 1, 2023), indicated by a gray box in Figure 1; characteristics of this cohort are summarized in the Table. eFigure 1 in Supplement 1 illustrates state-by-state proportions of individuals reporting high levels of trust (“a lot”) and low levels of trust (“not at all” or “a little”).

**Table.** Characteristics of Individuals With High or Low Levels of Trust in Physicians and Hospitals, Spring and Summer 2023^a^

Characteristic	Individuals, No. (%)			*P* value
	Trust some or less (n = 29 295)	Trust a lot (n = 21 253)	Total (n = 50 548)	
Trust in physicians and hospitals				
Not at all	1352 (4.6)	0	1352 (2.7)	<.001
A little	4264 (14.6)	0	4264 (8.4)	
Some	23 679 (80.8)	0	23 679 (46.8)	
A lot	0	21 253 (100.0)	21 253 (42.0)	
Respondent age, mean (SD), y	46.3 (16.5)	49.7 (18.4)	47.7 (17.4)	<.001
Gender				
Female	20 070 (68.5)	12 281 (57.8)	32 351 (64.0)	<.001
Male	9225 (31.5)	8972 (42.2)	18 197 (36.0)	
Educational level				
Some high school or less	1129 (3.9)	503 (2.4)	1632 (3.2)	<.001
High school graduate	7173 (24.5)	4160 (19.6)	11 333 (22.4)	
Some college	7935 (27.1)	4918 (23.1)	12 853 (25.4)	
College degree	9958 (34.0)	8036 (37.8)	17 994 (35.6)	
Graduate degree	3100 (10.6)	3636 (17.1)	6736 (13.3)	
Income, $^b^				
<25 000	7024 (24.0)	3939 (18.5)	10 963 (21.7)	<.001
25 000 to <50 000	8008 (27.3)	5185 (24.4)	13 193 (26.1)	
50 000 to <100 000	9335 (31.9)	6888 (32.4)	16 223 (32.1)	
≥100 000	4924 (16.8)	5232 (24.6)	10 156 (20.1)	
Race and ethnicity				
Asian American	1008 (3.4)	813 (3.8)	1821 (3.6)	<.001
Black	3605 (12.3)	2049 (9.6)	5654 (11.2)	
Hispanic	2852 (9.7)	1784 (8.4)	4636 (9.2)	
Native American	341 (1.2)	208 (1.0)	549 (1.1)	
Pacific Islander	382 (1.3)	227 (1.1)	609 (1.2)	
White	20 486 (69.9)	15 874 (74.7)	36 360 (71.9)	
Other^c^	621 (2.1)	298 (1.4)	919 (1.8)	
Urbanicity				
Rural	6402 (21.9)	3563 (16.8)	9965 (19.7)	<.001
Suburban	16 240 (55.4)	12 034 (56.6)	28 274 (55.9)	
Urban	6653 (22.7)	5656 (26.6)	12 309 (24.4)	
Trust in science^d^				
Not at all	1882 (6.4)	116 (0.5)	1998 (4.0)	<.001
A little	5594 (19.1)	826 (3.9)	6420 (12.7)	
Some	17 300 (59.1)	7241 (34.1)	24 541 (48.6)	
A lot	4475 (15.3)	13 041 (61.4)	17 516 (34.7)	
Trustworthiness of other people, mean (SD)^e^^,^^f^	4.8 (2.3)	6.0 (2.3)	5.3 (2.4)	<.001
Political affiliation^g^				
Democrat	8968 (30.7)	9239 (43.5)	18 207 (36.1)	<.001
Independent or other	11 976 (41.0)	6695 (31.6)	18 671 (37.0)	
Republican	8262 (28.3)	5283 (24.9)	13 545 (26.9)	
SARS-CoV-2 vaccination	19 800 (67.6)	18 204 (85.7)	38 004 (75.2)	<.001
SARS-CoV-2 booster	11 827 (40.4)	12 681 (59.7)	24 508 (48.5)	<.001
Influenza vaccination^h^	6455 (45.7)	6934 (68.8)	13 389 (55.3)	<.001

^a^ Sociodemographic characteristics of participants surveyed in spring and summer 2023, which reflects wave 27 (April 5 to May 5, 2023) and wave 28 (June 29 to August 1, 2023).

^b^ Income is missing for 13 participants.

^c^ Other race or ethnicity refers to individuals who selected the “Other” category from a checklist.

^d^ Trust in science is missing for 73 participants.

^e^ General trust is missing for 1307 participants.

^f^ Scale, 1 to 10 (1 indicates a lesser sense of general trustworthiness and 10 indicates that the survey respondent feels that most people can be trusted).

^g^ Political affiliation is missing for 125 participants.

^h^ Influenza vaccination status is unavailable for 26 355 participants, as it was collected only in wave 27.

**Figure 2.**
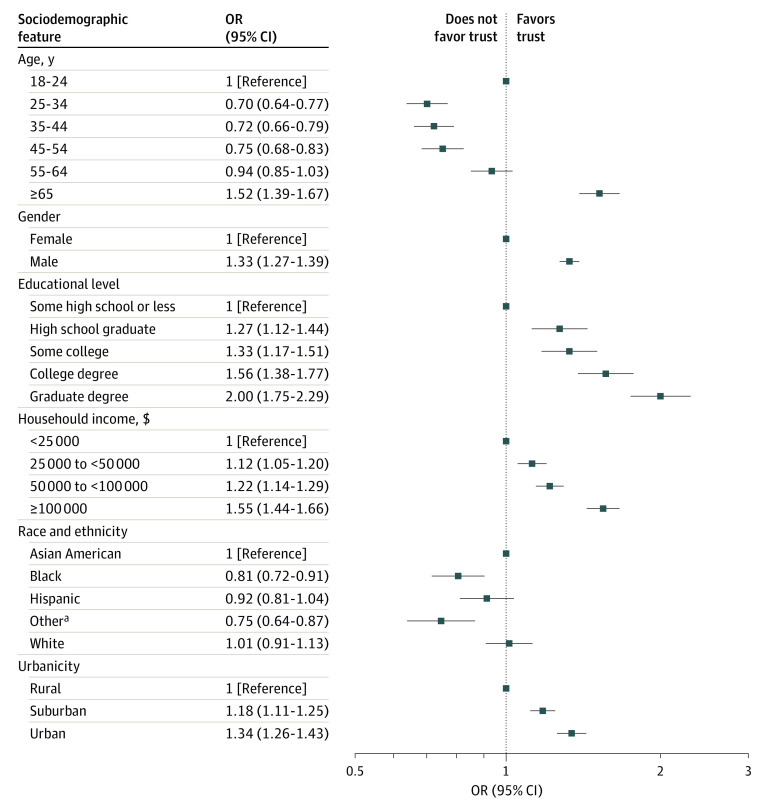
Association Between Individual Sociodemographic Features and Trust in Physicians and Hospitals in Ordinal Regression Models in Spring and Summer 2023 (N = 50 355) OR indicates odds ratio. ^a^Other race and ethnicity refers to individuals who indicated Native American, Pacific Islander, or “Other” from a survey checklist.

In these 2 waves, we examined associations between individual sociodemographic features and levels of trust in physicians and hospitals in ordinal regression models (Figure 2). Characteristics independently associated with decreased trust included being 25 to 64 years of age, female gender, lower educational level, lower income, Black race, and living in a rural area. Adding self-reported political affiliation did not meaningfully change these associations (eFigure 2 in Supplement 1).

**Figure 3.**
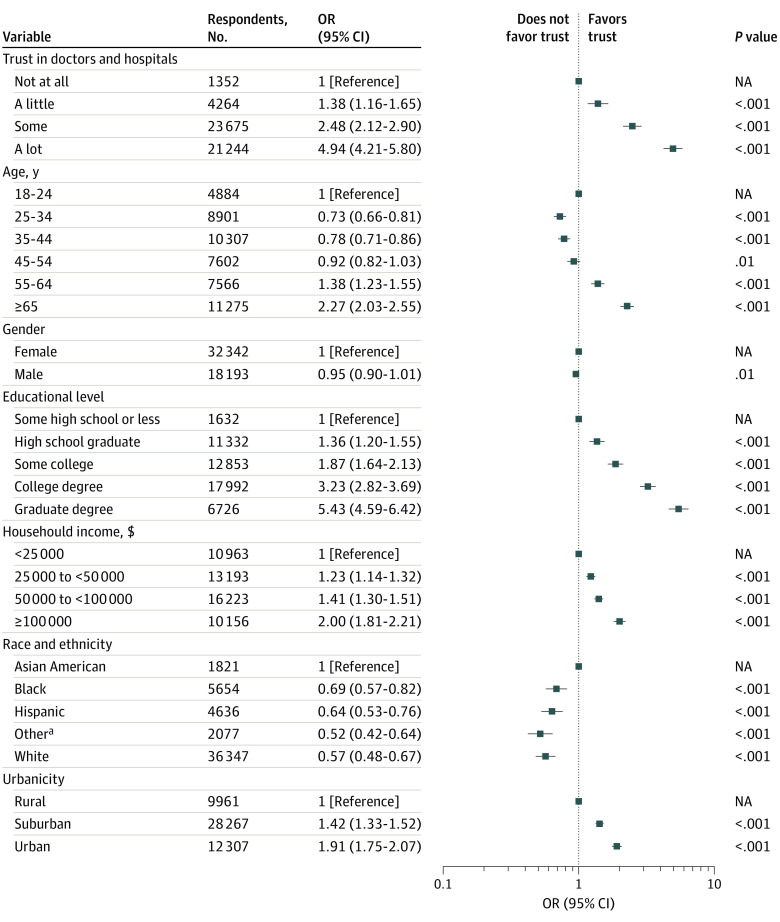
Association Between Trust in Physicians and Hospitals and SARS-CoV-2 Vaccination Status in Spring and Summer 2023, Adjusted for Sociodemographic Features NA indicates not applicable; OR, odds ratio. ^a^Other race and ethnicity refers to individuals who indicated Native American, Pacific Islander, or “Other” from a survey checklist.

We next examined the association between trust and COVID-19 vaccination status during these 2 waves. In logistic regression models, higher levels of trust were associated with a greater likelihood of being vaccinated in unadjusted models (a little trust vs none: odds ratio [OR], 1.63 [95% CI, 1.40-1.90]; some trust vs none: OR, 3.38 [95% CI, 2.95-3.88]; and a lot of trust vs none: OR, 7.59 [95% CI, 6.59-8.75]) and models adjusted for sociodemographic features (a little trust vs none: OR, 1.38 [95% CI, 1.16-1.65]; some trust vs none: OR, 2.48 [95% CI, 2.12-2.90]; and a lot of trust vs none: OR, 4.94 [95% CI, 4.21-5.80]) (Figure 3). Associations were not meaningfully different with further inclusion of political affiliation (eFigure 3 in Supplement 1).

Results were similar when considering SARS-CoV-2 vaccine boosters as the outcome, rather than any vaccination, in unadjusted models (a little trust vs none: OR, 1.54 [95% CI, 1.27-1.86]; some trust vs none: OR, 3.29 [95% CI, 2.77-3.92]; and a lot of trust vs none: OR, 5.96 [95% CI, 5.02-7.09]) and adjusted models (a little trust vs none: OR, 1.23 [95% CI, 1.00-1.52]; some trust vs none: OR, 2.22 [95% CI, 1.84-2.68]; and a lot of trust vs none: OR, 3.62 [95% CI, 2.99-4.38]) (eFigure 4 in Supplement 1). Inclusion of party affiliation was associated with similar results (eFigure 5 in Supplement 1).

We then repeated these analyses for influenza vaccination, available in the first of the 2 waves, to assess whether trust generalized beyond COVID-19 to other health-related behaviors. Once again, higher levels of trust were significantly associated with vaccination status in unadjusted models (a little trust vs none: OR, 1.40 [95% CI, 1.06-1.83]; some trust vs none: OR, 3.48 [95% CI, 2.72-4.46]; and a lot of trust vs none: OR, 7.43 [95% CI, 5.79-9.53]) and adjusted models (a little trust vs none: OR, 1.21 [95% CI, 0.91-1.61]; some trust vs none: OR, 2.63 [95% CI, 2.03-3.40]; and a lot of trust vs none: OR, 5.09 [95% CI, 3.93-6.59]) (eFigure 6 in Supplement 1). As in the other analyses, inclusion of political party affiliation yielded similar results (eFigure 7 in Supplement 1).

We also considered whether trust in physicians and hospitals was explained by other forms of trust, by adding overall sense of trustworthiness of other people, as well as trust in scientists, to the logistic regression model for vaccination status. Including these terms, greater trust in physicians and hospitals was significantly associated with SARS-CoV-2 vaccination (a little trust vs none: adjusted OR, 1.03 [95% CI, 0.84-1.26]; some trust vs none: adjusted OR, 1.37 [95% CI, 1.13-1.66]; and a lot of trust vs none: adjusted OR, 1.94 [95% CI, 1.59-2.36]) (eFigure 8 in Supplement 1).

**Figure 4.**
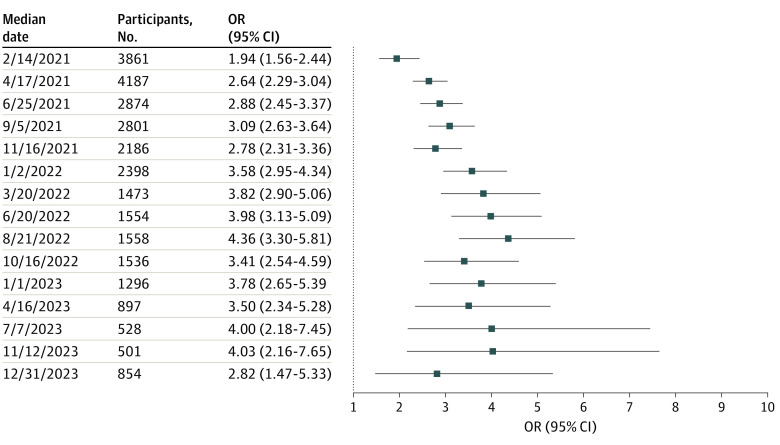
Association Between Trust in Physicians and Hospitals at Prior Survey Among Those Who Were Not Vaccinated and Likelihood of Becoming Vaccinated Against SARS-CoV-2 at Current Survey Wave, Adjusted for Sociodemographic Features Odds ratios (ORs) for vaccination associated with trust in physicians and hospitals at prior wave.

Although the survey design does not allow us to examine causation directly, we next considered lagged trust as a factor associated with vaccination status at the following wave, among individuals who responded to 2 consecutive waves. We estimated a logistic regression model at each survey wave beginning with wave 16 (January 2021), each with SARS-CoV-2 vaccination status as outcome and trust in physicians and hospitals at the preceding wave, along with sociodemographic features as in the prior models (Figure 4). At all time points, a high level of trust in physicians and hospitals was associated with greater odds of becoming vaccinated by the next wave; adjusted ORs ranged from 1.94 (95% CI, 1.56-2.44) in January 2021 to 4.36 (95% CI, 3.30-5.81) in August 2022.

Finally, to inform future interventions aimed at restoring trust, we examined open-ended responses collected from a randomly selected subset of participants in 1 survey wave (wave 27, the first of the 2 examined cross-sectionally) when individuals were asked to explain briefly why they had indicated a particular level of trust. Responses among the 2 lowest levels of trust (n = 200) included the following themes: financial motives over patient care (70 respondents [35.0%]), poor quality of care and negligence (55 respondents [27.5%]), other (39 respondents [19.5%]), influence of external entities and agendas (27 respondents [13.5%]), and discrimination and bias (9 respondents [4.5%]). Examples of each of these are reported in eAppendices 1 and 2 in Supplement 1.

## Discussion

Among more than half a million survey responses from US adults between April 2020 and January 2024, we found that trust in physicians and hospitals decreased throughout the COVID-19 pandemic across all sociodemographic groups. A lower level of trust was associated with decreased likelihood of vaccination against SARS-CoV-2 as well as influenza; these associations were not explained by political affiliation, nor fully accounted for by trust in science, suggesting some specificity for medicine per se.

The association that we observed with greater vaccination rates is also consistent with a history of literature associating other health outcomes with greater trust. A meta-analysis identified 47 such studies, with trust in physicians associated significantly with greater self-reported but not objective outcomes.^18^ With regard to COVID-19, a survey of approximately 3000 registered voters in North Dakota in April 2021 found an association between trust in physicians, as well as government, and SARS-CoV-2 vaccine uptake.^19^ A subsequent survey of 211 older adults found an association between trust in physicians and booster uptake as well.^20^

On the other hand, the change in trust during the pandemic may be specific to the US; prior studies suggested wide variation in levels of trust between countries before the pandemic,^21^ complicating any cross-national comparisons. Still, a large Chinese study found that trust increased markedly over the course of their COVID-19 response.^22^ In that country, trust in physicians had been diminished prior to the pandemic, and physicians were seen in national public health messaging as leading the fight against the pandemic.^22^

Despite the observed decrease in trust in US physicians during the pandemic, aggregate levels of trust in physicians and hospitals were still substantial. A prior cross-sectional survey of 4208 US adults in 2022 found greater trust in receiving health information from physicians and nurses than from all other US institutions.^5^ Our results are also consistent with results from smaller national probabilistic surveys—for example, a pilot Kaiser Family Foundation survey in May and June 2023 found that 48 of respondents reported a great deal of trust in physicians for health recommendations,^6^ but did not describe subgroup differences. Other panel-based surveys also identified a reduction in trust; a national panel of 2069 US adults surveyed in December 2020 and January 2021 found that 32% reported diminished trust in the health system during the pandemic.^23^ Likewise, a Gallup poll found that the proportion of individuals reporting trust in the health system had decreased from a high of 77% to 56% in 2023.^7^

Our results cannot establish causation, but in the context of prior studies documenting associations between physician trust and more positive health outcomes, they raise the possibility that the decrease in trust during the pandemic could have long-lasting public health implications. If so, effective interventions aimed at restoring trust could have benefits, not only for future pandemics, but for health in the US more generally, at least in terms of vaccination. In examining reasons for low trust, financial conflicts of interest, a longstanding area of academic investigation^24,25,26,27^ in medicine, remain a major factor associated with mistrust, concerns that may have been amplified during the pandemic. However, a prior Cochrane review concluded that there was a lack of evidence that any intervention meaningfully changed trust in physicians,^28^ despite a number of efforts to do so that observed generally modest effects. A better understanding of groups exhibiting particularly low trust, and the factors associated with that diminished trust, may be valuable in guiding future intervention development and deployment.

### Limitations

This study has multiple limitations. First, our assessment of trust relies on a single item, consistent with most other national surveys. Trust has been recognized to be a complex construct,^29,30,31^ with one early study identifying 9 domains related to trust in physicians.^32^ More nuanced understandings of trust may require use of multi-item scales, as a recent review suggested.^33^ Such scales may be particularly valuable in characterizing outcomes of interventions aimed at improving trust. Although the specific question used to assess trust changed in the midpoint of the study, the trend toward diminishing trust was consistent before and after this change; moreover, answers to the 2 questions were highly correlated within individuals. Moreover, as our question about trust asked about physicians and hospitals, comparisons with surveys that inquire only about physicians should be interpreted with caution, given other evidence that individuals have greater trust in physicians than in health systems.^23^ Likewise, we cannot generalize to other health care professionals.

A further limitation is the lack of a true panel design; while respondents could return for more than 1 survey, facilitating our lagged analysis, most participants in any given wave were not participants in the prior wave, and those who returned were not randomly sampled. The nonprobability sampling method has been criticized more generally for yielding less representative samples.^34^ On the other hand, prior validation efforts with the present survey, which incorporates quotas and attention checks to maximize representation and data quality, suggest a high degree of concordance with the traditional criterion standard methods.^13,14^ The survey was also administered in English, which may have led us to undersample underserved populations in the US with limited English proficiency or low literacy. Furthermore, the broad nature of the survey precluded assessment of health-specific characteristics (eg, insurance status, health care utilization, medical comorbidities) that may also influence trust. Finally, as we have noted, we can only examine associations with trust; we cannot determine if the associations we observe between trust and behavior are causal. Although a strength of our approach is the ability to control for multiple potential confounding variables, we likewise cannot exclude all potential confounders.

## Conclusions

Despite these caveats, this multiwave nationally representative survey identifies a substantial decrease in trust in physicians and hospitals during the COVID-19 pandemic and demonstrates associations between trust and health-related behavior after accounting for a host of potential confounding variables. Whether interventions to restore trust could increase compliance with vaccination and other positive health behaviors merits further investigation. In particular, our analyses of open-ended results suggest that factors associated with mistrust are heterogeneous, which may require more targeted interventions.

## Notes

Supplement 1. **eTable 1.** Date Ranges and Total Number of Respondents for Each Survey Wave **eTable 2.** Characteristics of Individuals With High or Low Levels of Trust Across 24 Survey Waves (April 2020–January 2024) **eFigure 1.** State-by-State Levels of Trust in Physicians and Hospitals in Spring/Summer 2023, Indicating High (Top) and Low or No (Bottom) Levels of Trust **eFigure 2.** Association Between Individual Sociodemographic Features and Trust in Physicians in Ordinal Regression Models, Including Self-Reported Partisan Affiliation **eFigure 3.** Association Between Trust and SARS-CoV-2 Vaccination Status in Spring/Summer 2023, With Addition of Partisan Affiliation **eFigure 4.** Association Between Trust and SARS-CoV-2 Booster Status in Spring/Summer 2023 **eFigure 5.** Association Between Trust and SARS-CoV-2 Booster Status in Spring/Summer 2023, With Addition of Partisan Affiliation **eFigure 6.** Association Between Trust and Influenza Vaccination Status in Spring/Summer 2023 **eFigure 7.** Association Between Trust and Influenza Vaccination Status in Spring/Summer 2023, With Addition of Partisan Affiliation **eFigure 8.** Association Between Trust and SARS-CoV-2 Vaccination Status in Spring/Summer 2023, With Addition of General Trust and Trust in Science **eFigure 9.** Proportion of Respondents Reporting Each Trust Level at Each Survey Wave **eAppendix 1.** Large Language Model Investigation of Themes in Free Text Responses Regarding Trust in Physicians and Hospitals **eAppendix 2.** Pertinent Survey Question Text

Supplement 2.
Data Sharing Statement
