# Supplementary material for: Trust in Physicians and Hospitals During the COVID-19 Pandemic in a 50-State Survey of US Adults
Source: JAMA Netw Open. 2024 Jul 31;7(7):e2424984. doi: 10.1001/jamanetworkopen.2024.24984 (PMC11292455; doi:10.1001/jamanetworkopen.2024.24984)
Supplement: Supplement 1. — eTable 1. Date Ranges and Total Number of Respondents for Each Survey Wave eTable 2. Characteristics of Individuals With High or Low Levels of Trust Across 24 Survey Waves (April 2020–January 2024) eFigure 1. State-by-State Levels of Trust in Physicians and Hospitals in Spring/Summer 2023, Indicating High (Top) and Low or No (Bottom) Levels of Trust eFigure 2. Association Between Individual Sociodemographic Features and Trust in Physicians in Ordinal Regression Models, Including Self-Reported Partisan Affiliation eFigure 3. Association Between Trust and SARS-CoV-2 Vaccination Status in Spring/Summer 2023, With Addition of Partisan Affiliation eFigure 4. Association Between Trust and SARS-CoV-2 Booster Status in Spring/Summer 2023 eFigure 5. Association Between Trust and SARS-CoV-2 Booster Status in Spring/Summer 2023, With Addition of Partisan Affiliation eFigure 6. Association Between Trust and Influenza Vaccination Status in Spring/Summer 2023 eFigure 7. Association Between Trust and Influenza Vaccination Status in Spring/Summer 2023, With Addition of Partisan Affiliation eFigure 8. Association Between Trust and SARS-CoV-2 Vaccination Status in Spring/Summer 2023, With Addition of General Trust and Trust in Science eFigure 9. Proportion of Respondents Reporting Each Trust Level at Each Survey Wave eAppendix 1. Large Language Model Investigation of Themes in Free Text Responses Regarding Trust in Physicians and Hospitals eAppendix 2. Pertinent Survey Question Text [file jamanetwopen-e2424984-s001.pdf]

Supplementary Online Content

Perlis RH, Ognyanova K, Uslu A, et al. Trust in physicians and hospitals during the COVID-19 pandemic in a 50-state survey of US adults. *JAMA Netw Open.* 2024;7(7):e2424984. doi:10.1001/jamanetworkopen.2024.24984

eTable 1. Date Ranges and Total Number of Respondents for Each Survey Wave
eTable 2. Characteristics of Individuals With High or Low Levels of Trust Across 24 Survey Waves (April 2020–January 2024)
eFigure 1. State-by-State Levels of Trust in Physicians and Hospitals in Spring/Summer 2023, Indicating High (Top) and Low or No (Bottom) Levels of Trust
eFigure 2. Association Between Individual Sociodemographic Features and Trust in Physicians in Ordinal Regression Models, Including Self-Reported Partisan Affiliation
eFigure 3. Association Between Trust and SARS-CoV-2 Vaccination Status in Spring/Summer 2023, With Addition of Partisan Affiliation
eFigure 4. Association Between Trust and SARS-CoV-2 Booster Status in Spring/Summer 2023
eFigure 5. Association Between Trust and SARS-CoV-2 Booster Status in Spring/Summer 2023, With Addition of Partisan Affiliation
eFigure 6. Association Between Trust and Influenza Vaccination Status in Spring/Summer 2023
eFigure 7. Association Between Trust and Influenza Vaccination Status in Spring/Summer 2023, With Addition of Partisan Affiliation
eFigure 8. Association Between Trust and SARS-CoV-2 Vaccination Status in Spring/Summer 2023, With Addition of General Trust and Trust in Science
eFigure 9. Proportion of Respondents Reporting Each Trust Level at Each Survey Wave
eAppendix 1. Large Language Model Investigation of Themes in Free Text Responses Regarding Trust in Physicians and Hospitals
eAppendix 2. Pertinent Survey Question Text

This supplementary material has been provided by the authors to give readers additional information about their work.

© 2024 Perlis RH et al. *JAMA Network Open.*

eTable 1. Date Ranges and Total Number of Respondents for Each Survey Wave

Wave	Start	End	N	Disqualified	Filtered	Note
1	4/16/2020	4/30/2020	21,405	16%	1%	
2	5/1/2020	5/21/2020	23,815	20%	1%	
3	5/16/2020	6/1/2020	20,391	24%	1%	
5	6/12/2020	6/28/2020	22,905	22%	1%	
7	7/10/2021	7/26/2020	19,437	19%	1%	
9	8/7/2020	8/26/2020	21,496	17%	1%	
10	9/4/2020	9/30/2020	23,061	15%	1%	
11	10/2/2020	10/23/2020	19,578	18%	3%	
13	11/3/2020	11/30/2020	26,655	21%	2%	
14	12/16/2020	2/8/2021	26,631	19%	2%	
16	2/5/2021	2/28/2021	23,364	19%	2%	
17	4/1/2021	5/3/2021	23,733	25%	2%	
18	6/9/2021	7/15/2021	22,286	22%	2%	
19	8/26/2021	11/3/2021	23,974	21%	2%	
20	11/3/2021	12/2/2021	24,626	27%	1%	
21	12/22/2021	2/8/2022	25,368	24%	1%	
22	3/2/2022	4/8/2022	23,380	27%	1%	
23	6/8/2022	8/1/2022	24,836	28%	1%	
24	8/11/2022	9/11/2022	26,559	25%	3%	
25	10/5/2022	11/9/2022	25,968	31%	1%	** wording of trust question changed
26	12/22/2022	1/17/2023	24,960	27%	2%	
27	4/4/2023	5/5/2023	24,267	26%	3%	** used in regression models
28	6/29/2023	8/1/2023	27,962	30%	2%	** used in regression models
29	11/2/2023	12/4/2023	28486	27%	1%	
30	12/21/2023	1/29/2024	30455	26%	2%	

Note: discontinuity in wave numbers reflects deployment of smaller surveys (e.g., wave 3, 4, and 6) without state-level data, not included in these analyses. 'Disqualified' refers to failing attention checks during the survey, resulting in immediate discontinuation; 'Filtered' refers to failing subsequent data quality checks, resulting in removal from data set after completion.

© 2024 Perlis RH et al. *JAMA Network Open.*

eTable 2. Characteristics of Individuals With High or Low Levels of Trust Across 24 Survey Waves (April 2020–January 2024)

Characteristic	Trust some or less (N=201618)	Trust a lot (N=241837)	Total (N=443455)
**Trust in doctors and hospitals**			
Not at all	10590 (5.3%)	0 (0.0%)	10590 (2.4%)
A little	28330 (14.1%)	0 (0.0%)	28330 (6.4%)
Some	162698 (80.7%)	0 (0.0%)	162698 (36.7%)
A lot	0 (0.0%)	241837 (100.0%)	241837 (54.5%)
**Respondent age (in years)**			
Mean (SD)	42.1 (15.5)	44.2 (17.3)	43.3 (16.6)
**Gender**			
Female	137741 (68.3%)	150445 (62.2%)	288186 (65.0%)
Male	63877 (31.7%)	91392 (37.8%)	155269 (35.0%)
**Education**			
Some High School or Less	8869 (4.4%)	7130 (2.9%)	15999 (3.6%)
High School Graduate	49864 (24.7%)	45125 (18.7%)	94989 (21.4%)
Some College	55702 (27.6%)	59290 (24.5%)	114992 (25.9%)
College Degree	66226 (32.8%)	87327 (36.1%)	153553 (34.6%)
Graduate Degree	20957 (10.4%)	42965 (17.8%)	63922 (14.4%)
**Income (a)**			
Under $25K	52434 (26.4%)	48050 (20.4%)	100484 (23.1%)
$ 25k to under $50k	53977 (27.2%)	56190 (23.8%)	110167 (25.4%)
$ 50K to under $100K	60548 (30.5%)	76073 (32.3%)	136621 (31.5%)
$100K and over	31613 (15.9%)	55407 (23.5%)	87020 (20.0%)
**Race and Ethnicity**			
Asian American	8662 (4.3%)	13295 (5.5%)	21957 (5.0%)
Black	26154 (13.0%)	23274 (9.6%)	49428 (11.1%)
Hispanic	18986 (9.4%)	19437 (8.0%)	38423 (8.7%)
Native American	1615 (0.8%)	1523 (0.6%)	3138 (0.7%)
Other (b)	5256 (2.6%)	4377 (1.8%)	9633 (2.2%)
Pacific Islander	2955 (1.5%)	2643 (1.1%)	5598 (1.3%)
White	137990 (68.4%)	177288 (73.3%)	315278 (71.1%)
**Urbanicity**			
Rural	44850 (22.2%)	41653 (17.2%)	86503 (19.5%)
Suburban	113212 (56.2%)	139808 (57.8%)	253020 (57.1%)
Urban	43556 (21.6%)	60376 (25.0%)	103932 (23.4%)

(a) Household income missing for n=9163 individuals (3046 and 6117, respectively); (b) Other race and ethnicity refers to individuals who selected 'Other' from a checklist of options.

© 2024 Perlis RH et al. *JAMA Network Open.*

eFigure 1. State-by-State Levels of Trust in Physicians and Hospitals in Spring/Summer 2023, Indicating High (Top) and Low or No (Bottom) Levels of Trust

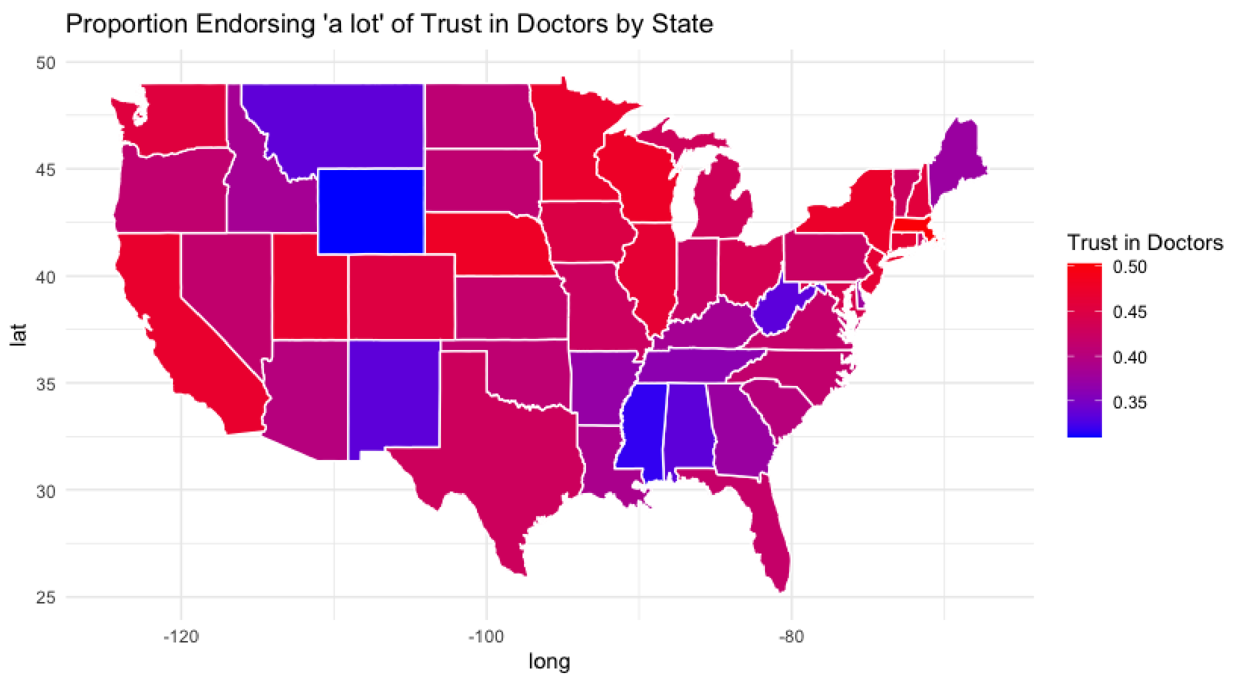

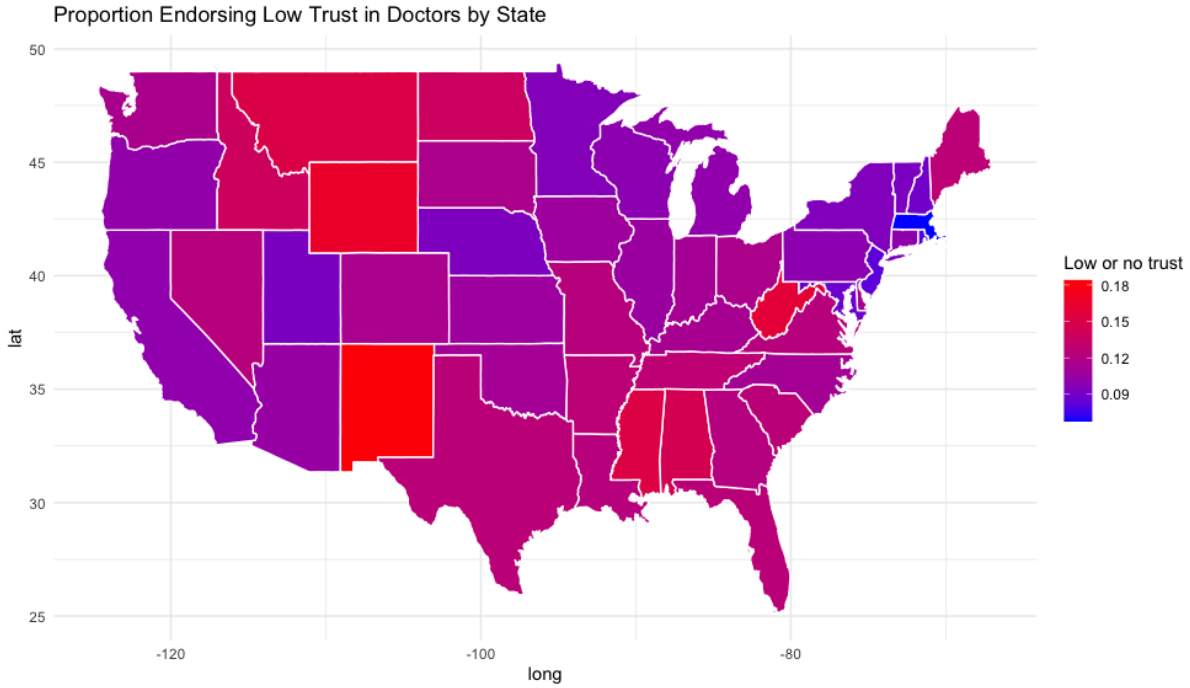

© 2024 Perlis RH et al. *JAMA Network Open.*

eFigure 2. Association Between Individual Sociodemographic Features and Trust in Physicians in Ordinal Regression Models, Including Self-Reported Partisan Affiliation

Characteristic	Level	OR	95% CI
**Age**	18 to 24	1.00	
	25 to 34	0.70	0.64, 0.77
	35 to 44	0.71	0.65, 0.77
	45 to 54	0.75	0.69, 0.83
	55 to 64	0.93	0.84, 1.02
	65 and over	1.49	1.36, 1.64
**Gender**	Female	1.00	
	Male	1.37	1.31, 1.43
**Education**	Some High School or Less	1.00	
	High School Graduate	1.23	1.09, 1.40
	Some College	1.27	1.12, 1.44
	College Degree	1.44	1.27, 1.64
	Graduate Degree	1.77	1.55, 2.03
**Income**	Under 25K	1.00	
	25k to under 50k	1.12	1.05, 1.19
	50K to under 100K	1.22	1.14, 1.30
	100K and over	1.51	1.40, 1.63
**Race and Ethnicity**	Asian American	1.00	
	Black	0.73	0.64, 0.82
	Hispanic	0.87	0.77, 0.99
	Other	0.76	0.65, 0.89
	White	1.03	0.93, 1.15
**Urbanicity**	Rural	1.00	
	Suburban	1.14	1.08, 1.21
	Urban	1.23	1.15, 1.31
**Political affiliation**	Democrat	1.00	
	Independent/Other	0.54	0.52, 0.57
	Republican	0.58	0.55, 0.61

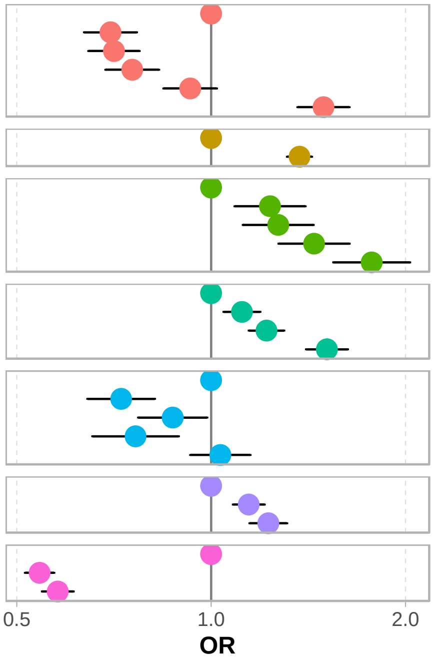

© 2024 Perlis RH et al. *JAMA Network Open.*

eFigure 3. Association Between Trust and SARS-CoV-2 Vaccination Status in Spring/Summer 2023, With Addition of Partisan Affiliation

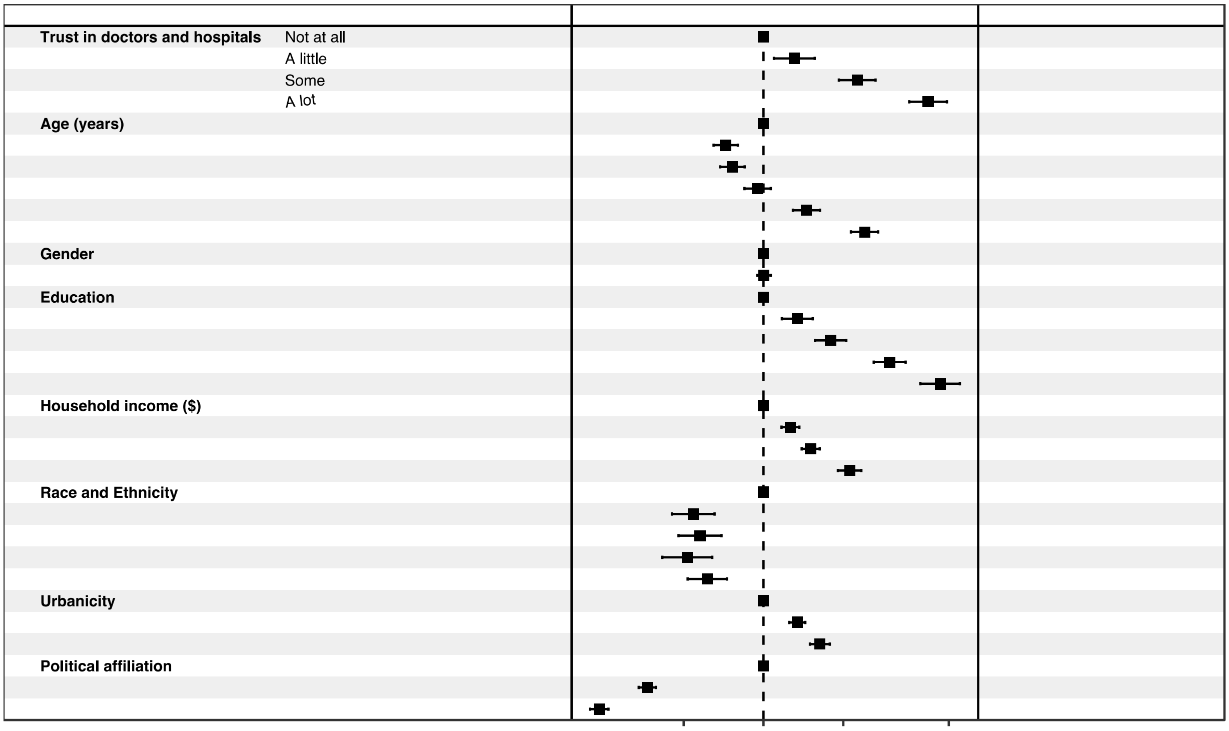

© 2024 Perlis RH et al. *JAMA Network Open.*

eFigure 4. Association Between Trust and SARS-CoV-2 Booster Status in Spring/Summer 2023

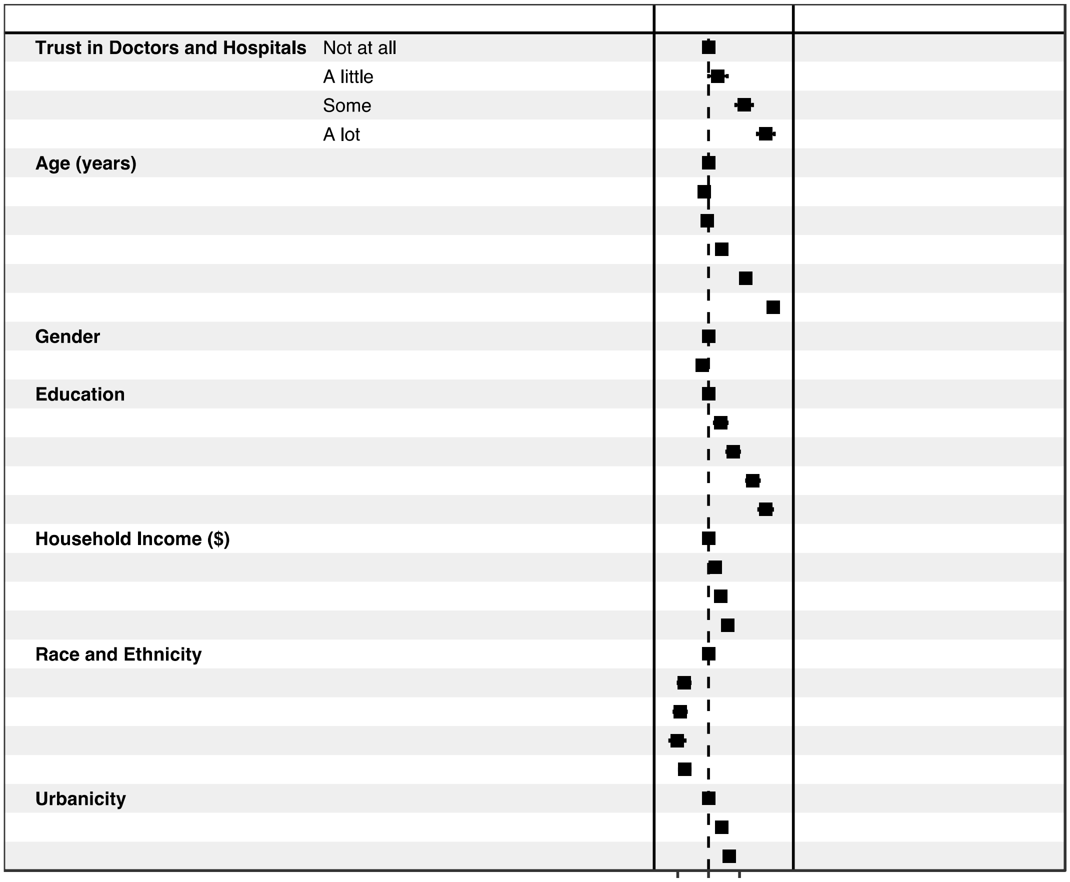

© 2024 Perlis RH et al. *JAMA Network Open.*

eFigure 5. Association Between Trust and SARS-CoV-2 Booster Status in Spring/Summer 2023, With Addition of Partisan Affiliation

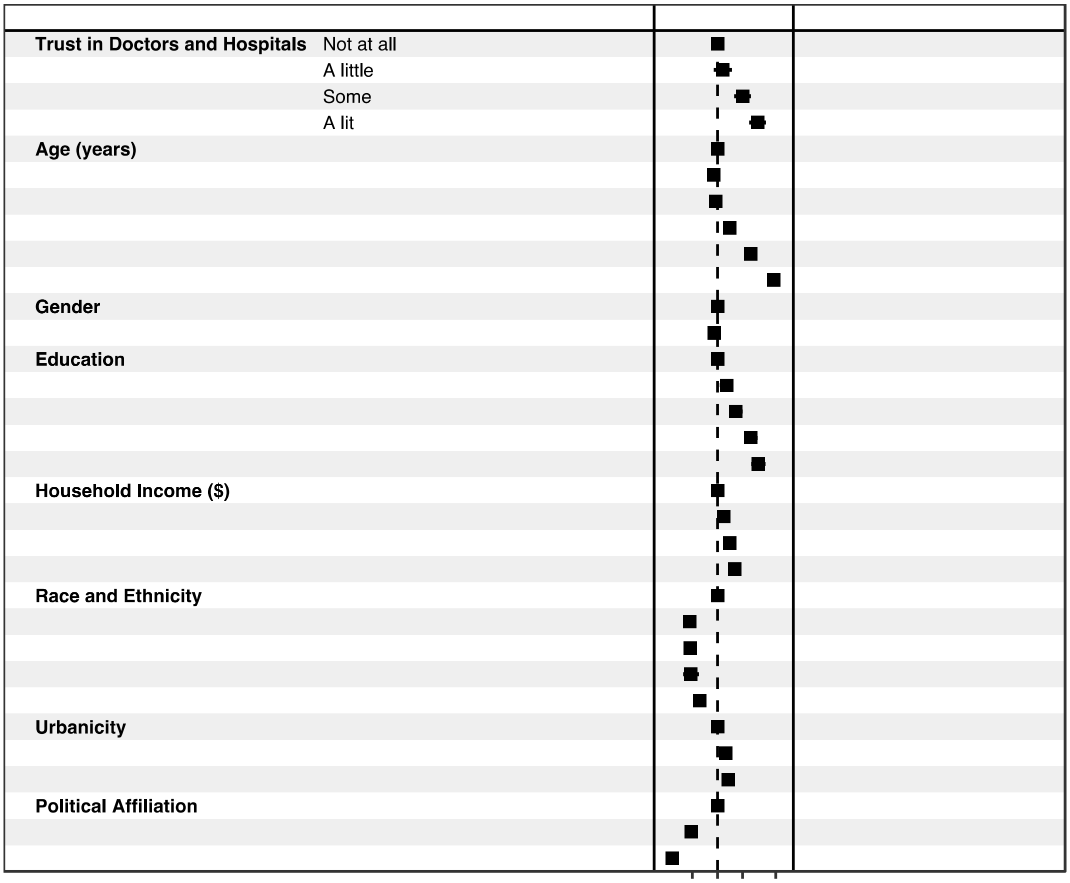

© 2024 Perlis RH et al. *JAMA Network Open.*

eFigure 6. Association Between Trust and Influenza Vaccination Status in Spring/Summer 2023

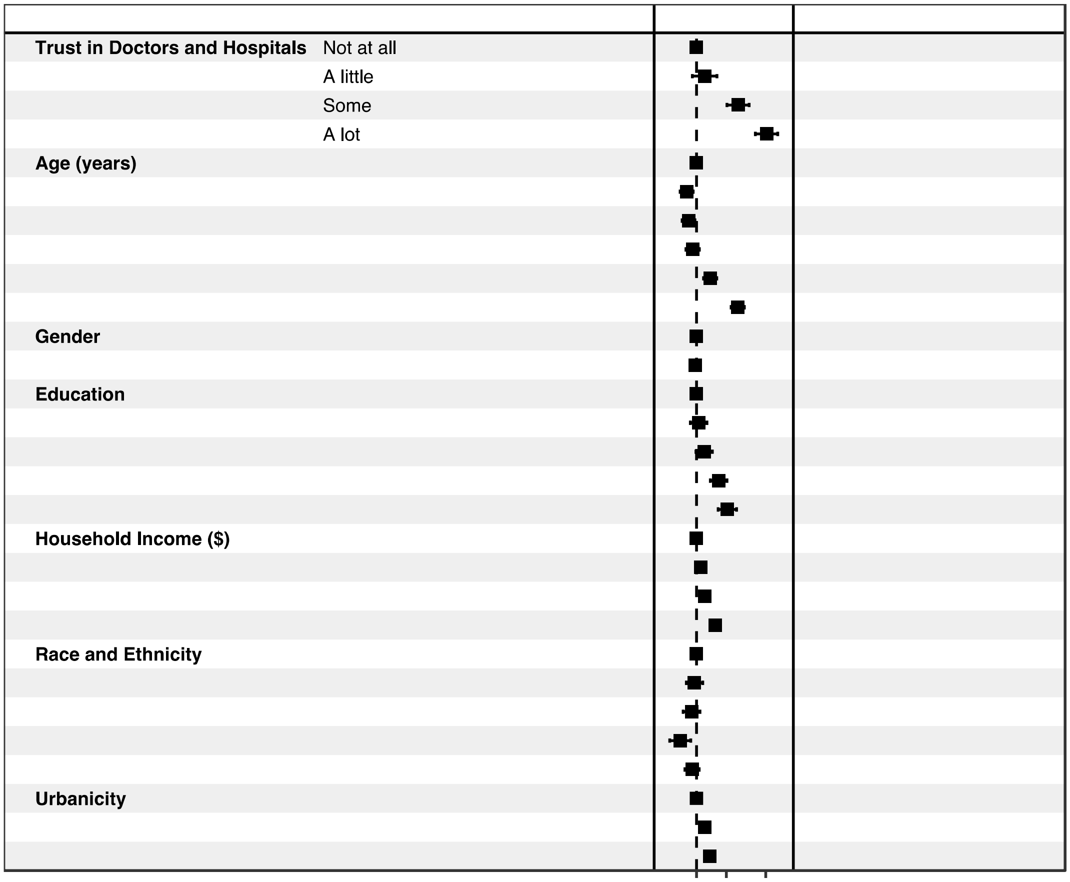

© 2024 Perlis RH et al. *JAMA Network Open.*

eFigure 7. Association Between Trust and Influenza Vaccination Status in Spring/Summer 2023, With Addition of Partisan Affiliation

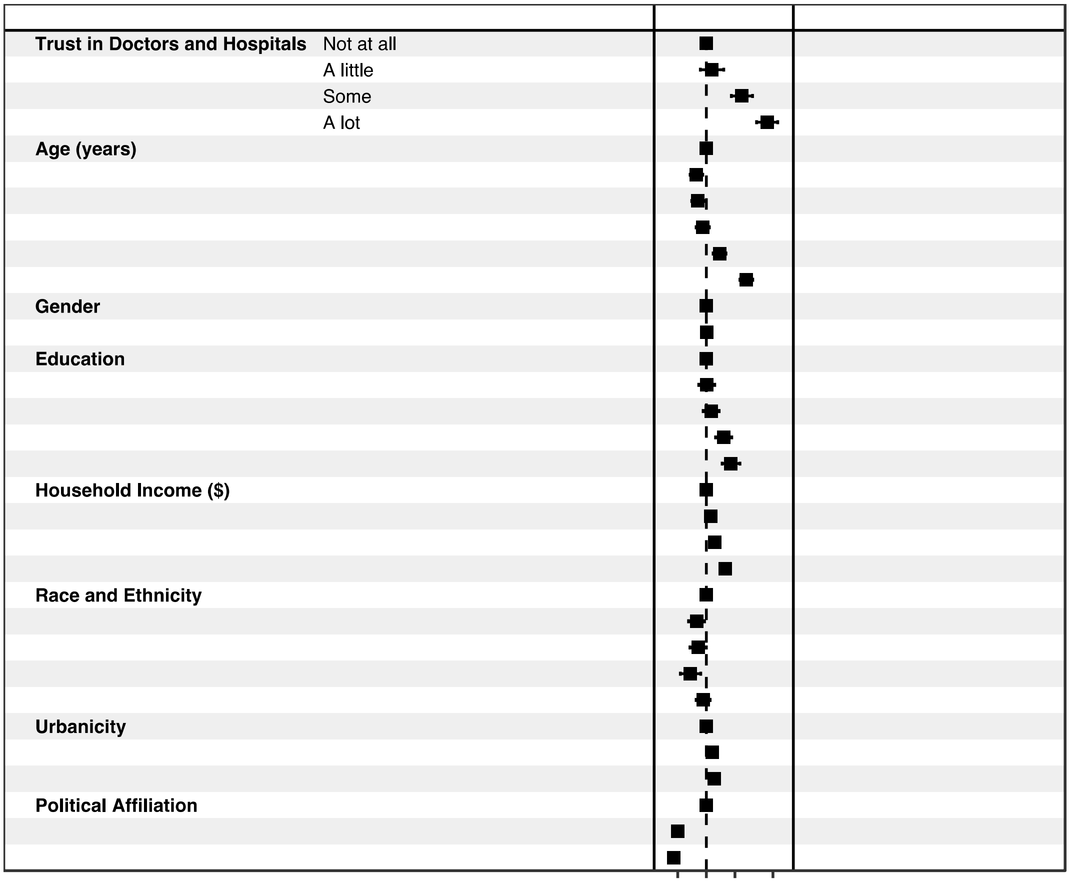

© 2024 Perlis RH et al. *JAMA Network Open.*

eFigure 8. Association Between Trust and SARS-CoV-2 Vaccination Status in Spring/Summer 2023, With Addition of General Trust and Trust in Science

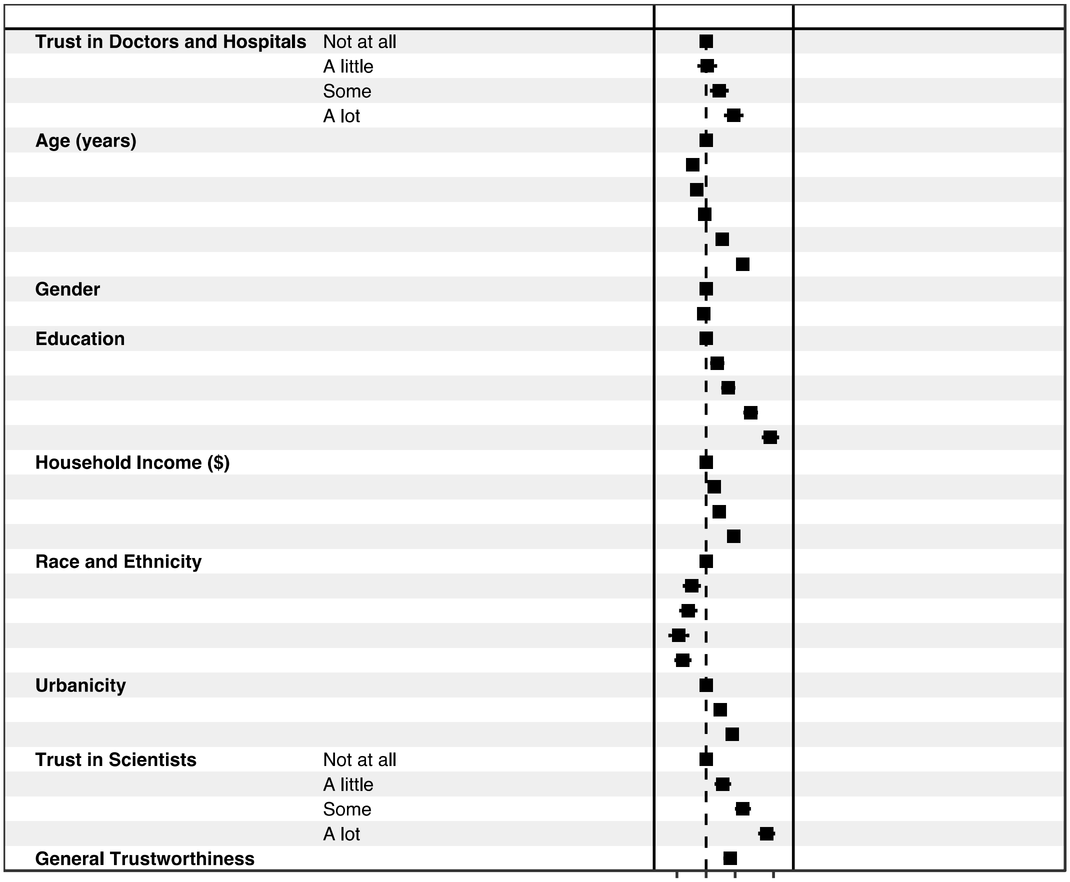

© 2024 Perlis RH et al. *JAMA Network Open.*

eFigure 9. Proportion of Respondents Reporting Each Trust Level at Each Survey Wave

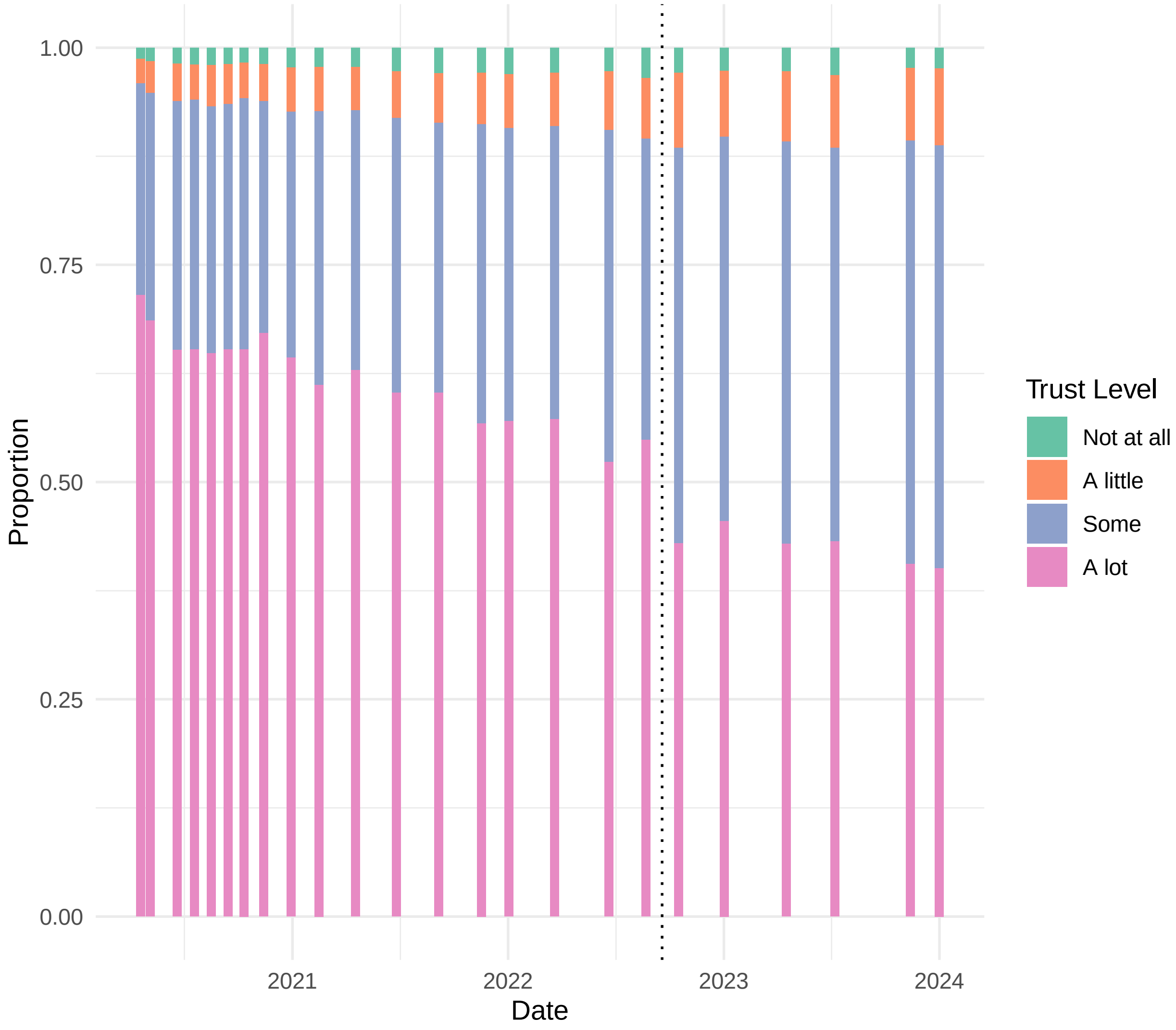

Note: Dotted line indicates change in wording of survey question regarding trust; see text for details.

© 2024 Perlis RH et al. *JAMA Network Open.*

eAppendix 1. Large Language Model Investigation of Themes in Free Text Responses Regarding Trust in Physicians and Hospitals

Model output:

Based on the survey responses provided, we can identify four main themes and an 'other' category for comments that don't fit into these themes. The themes reflect common concerns and experiences related to distrust in doctors and hospitals:

1. **Financial Motives Over Patient Care**: This theme includes perceptions of healthcare as primarily profit-driven, where financial incentives outweigh patient welfare. Respondents believe that decisions are made based on profitability rather than the best interests of patients.

2. **Poor Quality of Care and Negligence**: Responses that mention experiences of neglect, inadequate care, misdiagnosis, or dismissive attitudes from healthcare providers fall under this category. This also includes perceptions of healthcare professionals not listening or taking patient concerns seriously.

3. **Influence of External Entities and Agendas**: Here, the focus is on the belief that decisions in healthcare are unduly influenced by pharmaceutical companies, government entities, or other external powers. This includes suspicions of dishonesty or withholding information for non-medical reasons.

4. **Discrimination and Bias**: Responses indicating experiences or beliefs that healthcare providers exhibit bias, discrimination, or lack of cultural competency. This can include racial discrimination, gender bias, or insensitivity to patient backgrounds.

5. **Other**: Any comments that do not clearly fit into the themes above, including vague or nonspecific distrust, personal beliefs, or unique experiences not shared by other respondents.

Financial Motives Over Patient Care
1. "Doctors and hospitals are one of the biggest multi million dollar industry..."

2. "It seems they only do things for money and to keep their jobs."
3. "They're all interested in profits over patients."
4. "Health last money first."

Poor Quality of Care and Negligence
1. "They have not treated me well."
2. "They didn’t take care of my son in law like they should..."
3. "Often miss diagnos"
4. "It's more the nurses, negligence from them is only rising."

Influence of External Entities and Agendas
1. "They've been denying healthcare to those who refused the covid jab..."
2. "The media makes them tell us what they need us to hear."
3. "Because they are told what to tell us."
4. "I believe they are all controlled by a higher rank of power..."

Discrimination and Bias
1. "Not the best at doing their jobs at time due to bigotry."
2. "I have one parent who is African American and one parent who is indigenous American..."
3. "Black women die at an alarming rate from their practices."
4. "Because \"white\" and \"black\" people get treated differently and not in a good way."

Other
1. "Just don't."
2. "I don't trust people easily."
3. "No reason."
4. "Not sure."

© 2024 Perlis RH et al. *JAMA Network Open.*

eAppendix 2. Pertinent Survey Question Text

How much do you trust the following people and organizations to do what is right? - Hospitals and doctors
4 = A lot
3 = Some
2 = Not too much
1 = Not at all

How much do you trust the following people and organizations to do what is right? - Scientists and researchers
4 = A lot
3 = Some
2 = Not too much
1 = Not at all

You said you trust doctors and hospitals [amount of trust]. Can you tell us why that is?

Generally speaking, would you say that most people can be trusted, or that you cannot be too careful in dealing with people? Please give your answer on a scale from 1 to 10, where one is you cannot be too careful and 10 is most people can be trusted.

Have you ever received a COVID-19 vaccine?
1 = Yes, one dose
2 = Yes, two doses
4 = Yes, three doses
5 = Yes, four or more doses
3 = No

Have you or will you get a flu shot this season?

© 2024 Perlis RH et al. *JAMA Network Open.*

1 = Yes, I already got it

2 = Yes, I plan to get it

8 = Maybe, if I get around to it

3 = Not sure, thinking about it

4 = No, it is too difficult/expensive to get

5 = No, I worry it is bad for my health

6 = No, I don’t think it is effective

7 = No, for other reasons

© 2024 Perlis RH et al. *JAMA Network Open.*
