# Supplementary material for: Trust in Physicians and Hospitals During the COVID-19 Pandemic in a 50-State Survey of US Adults
Source: JAMA Netw Open. 2024 Jul 31;7(7):e2424984. doi: 10.1001/jamanetworkopen.2024.24984 (PMC11292455; doi:10.1001/jamanetworkopen.2024.24984)
Supplement: Supplement 2. — Data Sharing Statement [file jamanetwopen-e2424984-s002.pdf]

Data Sharing Statement

Perlis. Trust in Physicians and Hospitals During the COVID-19 Pandemic in a 50-State Survey of US Adults. *JAMA Netw Open*. Published July 31, 2024.
doi:10.1001/jamanetworkopen.2024.24984

Data
Data available: Yes
Data types: Deidentified participant data
How to access data: covidstates.org
When available: With publication

Supporting Documents
Document types: None

Additional Information
Who can access the data: researchers with approved proposal for data use
Types of analyses: for a specified purpose
Mechanisms of data availability: after approval of a proposal
